# Unintended pregnancy and associated factors among women on antiretroviral therapy in selected public hospitals, Addis Ababa, Ethiopia: A mixed-method study

**DOI:** 10.1371/journal.pgph.0007626

**Published:** 2026-11-12

**Authors:** Abraham Azene Ageze, Kasim Muhammed Yusuf, Andamlak Gizaw Alamdo, Gininu Wendmeneh Tsegaw

**Affiliations:** 1 Department of Public Health, Saint Paul’s Hospital Millennium Medical College, Addis Ababa, Ethiopia; 2 Department of Health Service Quality Improvement, Bella Health Center, Addis Ababa, Ethiopia; 3 Department of Health Service Quality Improvement, Menelik II Comprehensive Specialized Hospital, Addis Ababa, Ethiopia; PLOS: Public Library of Science, UNITED STATES OF AMERICA

## Abstract

Unintended pregnancy remains a public health concern among women living with HIV, despite expanded access to antiretroviral therapy (ART) and reproductive health services. Evidence on its prevalence and associated factors among women receiving ART in Ethiopia is limited. This study assessed the prevalence of unintended pregnancy and associated factors among women receiving ART in selected public hospitals in Addis Ababa, Ethiopia. A facility-based mixed-methods cross-sectional study was conducted among 384 women from June 12 to July 18, 2025. Quantitative data were collected using an interviewer-administered questionnaire and clinical record review. Participants were selected through systematic random sampling, while 14 participants were purposively selected for in-depth interviews. Quantitative data were analyzed using binary logistic regression, and qualitative data were analyzed thematically. The prevalence of unintended pregnancy was 38.8% (n = 149). Higher odds of unintended pregnancy were observed among women who did not use contraception after HIV diagnosis (AOR = 4.77; 95% CI: 2.85–8.04), had been diagnosed with HIV for ≥5 years (AOR = 2.77; 95% CI: 1.44–5.34), had inadequate sexual and reproductive health counseling (AOR = 1.86; 95% CI: 1.08–3.20), or had not disclosed their HIV status to partners (AOR = 1.92; 95% CI: 1.11–3.31). Compared with women with no formal education, those with primary, secondary, and higher education had lower odds of unintended pregnancy (AOR = 0.31; 95% CI: 0.17–0.56; AOR = 0.24; 95% CI: 0.11–0.51; and AOR = 0.11; 95% CI: 0.02–0.66, respectively). Qualitative findings highlighted fear of side effects, partner opposition, contraceptive misconceptions, inadequate counseling, and fragmented services as contributors to contraceptive non-use and unintended pregnancies. More than one-third of women receiving ART experienced unintended pregnancy, highlighting gaps in integrated reproductive health services. Strengthening family planning integration into ART care and improving reproductive counseling may help reduce unintended pregnancy.

## Introduction

Unintended pregnancy remains a major public health concern with significant implications for maternal and child health [1,2]. It is defined as a pregnancy that is either unwanted, occurring when no more children were desired, or mistimed, occurring earlier than desired [3]. Globally, an estimated 74 million unintended pregnancies occur annually in low- and middle-income countries, contributing to millions of unsafe abortions and substantial maternal morbidity and mortality [4]. The consequences are particularly serious among women living with HIV, who face additional clinical and social vulnerabilities.

Women of reproductive age continue to be disproportionately affected by HIV, particularly in Sub-Saharan Africa, where women and girls account for most new infections [5,6]. Increased biological susceptibility, gender-based violence, economic dependency, and restrictive social norms heighten women’s vulnerability [7,8]. In Ethiopia, women represent a substantial proportion of people living with HIV, and the estimated adult HIV prevalence in 2024 was 0.84% nationally, while Addis Ababa had a higher prevalence of 3.20% [9]. Spatial analyses have also demonstrated geographic variation in HIV distribution, with higher HIV prevalence observed in urban areas, including Addis Ababa [10]. These epidemiologic patterns demonstrate the importance of addressing reproductive health needs among women living with HIV in high-burden urban settings.

Preventing unintended pregnancy among women living with HIV is one of the four pillars of the World Health Organization’s Prevention of Mother-to-Child Transmission (PMTCT) strategy [11,12]. The second pillar specifically focuses on preventing unintended pregnancies among women living with HIV, recognizing that more than 90% of pediatric HIV infections result from vertical transmission during pregnancy, delivery, or breastfeeding [13]. Family planning is therefore recognized as a cost-effective intervention that reduces maternal morbidity and mortality and prevents pediatric HIV infection.

Despite expanded access to antiretroviral therapy (ART) and PMTCT services, unintended pregnancy remains common among women living with HIV. Globally, approximately 41% of pregnancies are unintended, and nearly half end in abortion [14]. Women living with HIV face a two- to tenfold higher risk of maternal mortality during pregnancy and the postpartum period compared with HIV-negative women [15]. HIV infection is also associated with increased risks of obstetric complications and HIV-related maternal deaths [15,16]. Unintended pregnancy may further compromise ART adherence and viral suppression, thereby increasing the risk of mother-to-child transmission [17].

Evidence indicates that unintended pregnancy is more prevalent among women living with HIV than among HIV-negative women [18]. Prevalence varies considerably across regions, ranging from 56% to 78% in North America and Europe [19–21], 63% to 82% in Latin America [22], and 37% to 71% in several African countries [23–25]. A recent systematic review and meta-analysis conducted in East Africa reported a pooled prevalence of 40.98% among women living with HIV, with a pooled prevalence of 28.38% in Ethiopia [15]. In Ethiopia, a facility-based study conducted in Addis Ababa reported a prevalence of 46% among women attending ART clinics [26]. These findings indicate that unintended pregnancy among women on ART remains a persistent public health challenge.

Multiple factors contribute to unintended pregnancy among women living with HIV. Younger age, single marital status, low education, and poor socioeconomic status increase vulnerability [18,27–29], while higher parity and prior abortion also elevate risk [20,25]. Contraceptive determinants—including nonuse, unmet need, method failure, and limited knowledge of dual protection—remain central [30,31]. Despite recommendations to integrate family planning into HIV care [32], implementation in Ethiopia remains limited [33,34], with only 43.6% of women receiving ART in Addis Ababa reporting current use of modern contraceptives, alongside persistent gaps in reproductive health counseling [35,36]. Clinical and behavioral factors such as lower CD4 count, higher viral load, longer ART duration, non-disclosure, and poor PMTCT knowledge, have also been associated with an increased risk of unintended pregnancy [25,27,37,38]. Overall, the existing literature indicates that both sociodemographic and contraceptive-related factors are commonly associated with unintended pregnancy among women living with HIV, whereas findings regarding HIV-related clinical characteristics have been less consistent across studies.

Despite national efforts to integrate family planning and HIV services, gaps persist in routine ART care, particularly in counseling quality and partner communication. A phenomenological qualitative study conducted in Addis Ababa explored the reasons for unintended pregnancy and prevention strategies from the perspectives of women and healthcare providers. However, evidence integrating quantitative and qualitative findings to estimate the prevalence, identify associated factors, and explore contextual experiences among women receiving ART remains limited. Quantitative studies alone may not fully capture the interpersonal, and health-system factors influencing reproductive decision-making among women living with HIV. Therefore, this mixed-methods study was conducted to address this gap by combining quantitative assessment of unintended pregnancy and associated factors with qualitative exploration of the experiences and perspectives of women, healthcare providers, and adherence supporters. This integrated approach provides evidence to better understand contraceptive practices, SRH counseling experiences, partner-related barriers, and opportunities for strengthening HIV and reproductive health service integration within ART clinics. Accordingly, this study assessed the prevalence and associated factors of unintended pregnancy among women attending ART clinics in public hospitals of Addis Ababa, Ethiopia, while also exploring contextual and service-related influences.

## Materials and methods

### Ethics statement

Ethical approval was obtained from the Institutional Review Board of Saint Paul’s Hospital Millennium Medical College, School of Public Health, and the study was conducted in accordance with the Declaration of Helsinki. Official permission was obtained from the Addis Ababa City Administration Health Bureau and the participating public hospitals before data collection. Written informed consent was obtained from all HIV-positive women before enrollment, and participation was voluntary, with participants having the right to withdraw at any time without consequences. Interviews were conducted in private settings, no personal identifiers were collected, and unique codes were used to maintain confidentiality. All data were stored securely and used solely for research purposes.

### Study area and period

The study was conducted among women receiving antiretroviral therapy (ART) in selected public hospitals in Addis Ababa, Ethiopia. Data were collected from 12 June to 18 July 2025. Addis Ababa is the capital city of Ethiopia and the country’s largest urban center. At the time of the study, 12 government hospitals administered by the Addis Ababa Regional Health Bureau and the Federal Ministry of Health were providing comprehensive HIV-related services, including ART, prevention of mother-to-child transmission (PMTCT), and family planning services. According to the Ethiopian Public Health Institute, more than 64,580 adult women were receiving ART in Addis Ababa during the study period [39–41].

### Study design

A mixed-method study was conducted using quantitative data collection complemented by qualitative in-depth interviews. The qualitative component employed a phenomenological approach to explore the sexual and reproductive health experiences of HIV-positive women. In-depth interviews were conducted with women who were not included in the quantitative survey but attended the same ART clinics.

### Population and study unit

The source population comprised all reproductive-age women attending ART units in selected public hospitals in Addis Ababa. The study population included reproductive-age women attending the selected ART clinics during the data collection period who met the eligibility criteria. The individual woman attending the ART clinic was considered the study unit.

### Eligibility criteria

#### Inclusion criteria.

Women were included if they were aged 15–49 years, had experienced at least one pregnancy after HIV diagnosis within the previous five years, and had attended the ART clinic at least once prior to data collection.

#### Exclusion criteria.

Women were excluded if they were severely ill, mentally unable to respond, or attending the ART clinic for the first time during the study period.

### Sample size and sampling procedure

#### Quantitative component.

**Sample size determination.** The sample size was determined based on the study's two objectives. For the first objective, which aimed to estimate the prevalence of unintended pregnancy, the sample size was determined using the single population proportion formula. Assuming a 46% prevalence among HIV-positive women [26], a 95% confidence level, and a 5% margin of error, the calculation was:

$n=\frac{{\left(Z_{a/\text{2}}\right)}^{\text{2}}p(\text{1}-p)}{d^{\text{2}}}=\frac{\left(\text{1}.\text{9}\text{6}{)}^{\text{2}}\text{0}.\text{4}\text{6}(\text{1}-\text{0}.\text{4}\text{6}\right)}{{\text{0}.\text{0}\text{5}}^{\text{2}}}=382$

After adding 10% to account for potential non-response, the final sample size for this objective was 421 participants. For the second objective, which aimed to identify factors associated with unintended pregnancy, sample size was calculated using Epi Info version 7.2 with the double population proportion formula, using the proportions of unintended pregnancy among the exposed and unexposed groups reported in a previous study, assuming a 95% confidence level, 80% power, a 1:1 ratio of exposed to unexposed, and 10% non-response. Key variables included non-use of contraception (56.3% vs. 32.6%, AOR = 2.65) and lack of knowledge on MTCT/PMTCT (56.1% vs. 29.2%, AOR = 3.1), which yielded sample sizes of 168 and 132, respectively [42]. The larger sample size obtained from the two objectives was adopted as the final sample size, resulting in 421 participants for the study.

**Sampling techniques and procedures.** Five ART treatment units were randomly selected from the 12 public hospitals in Addis Ababa. The sample was proportionally allocated to each hospital based on the number of reproductive-age women attending ART clinics during the study period (nᵢ = Nᵢ/3,822 × 421), resulting in an overall sampling fraction of approximately 0.11 (421/3,822). Systematic random sampling was used to select participants, with the first participant chosen by lottery and every k^th^ client thereafter until the required sample size was achieved (Fig 1).

**Fig 1:**
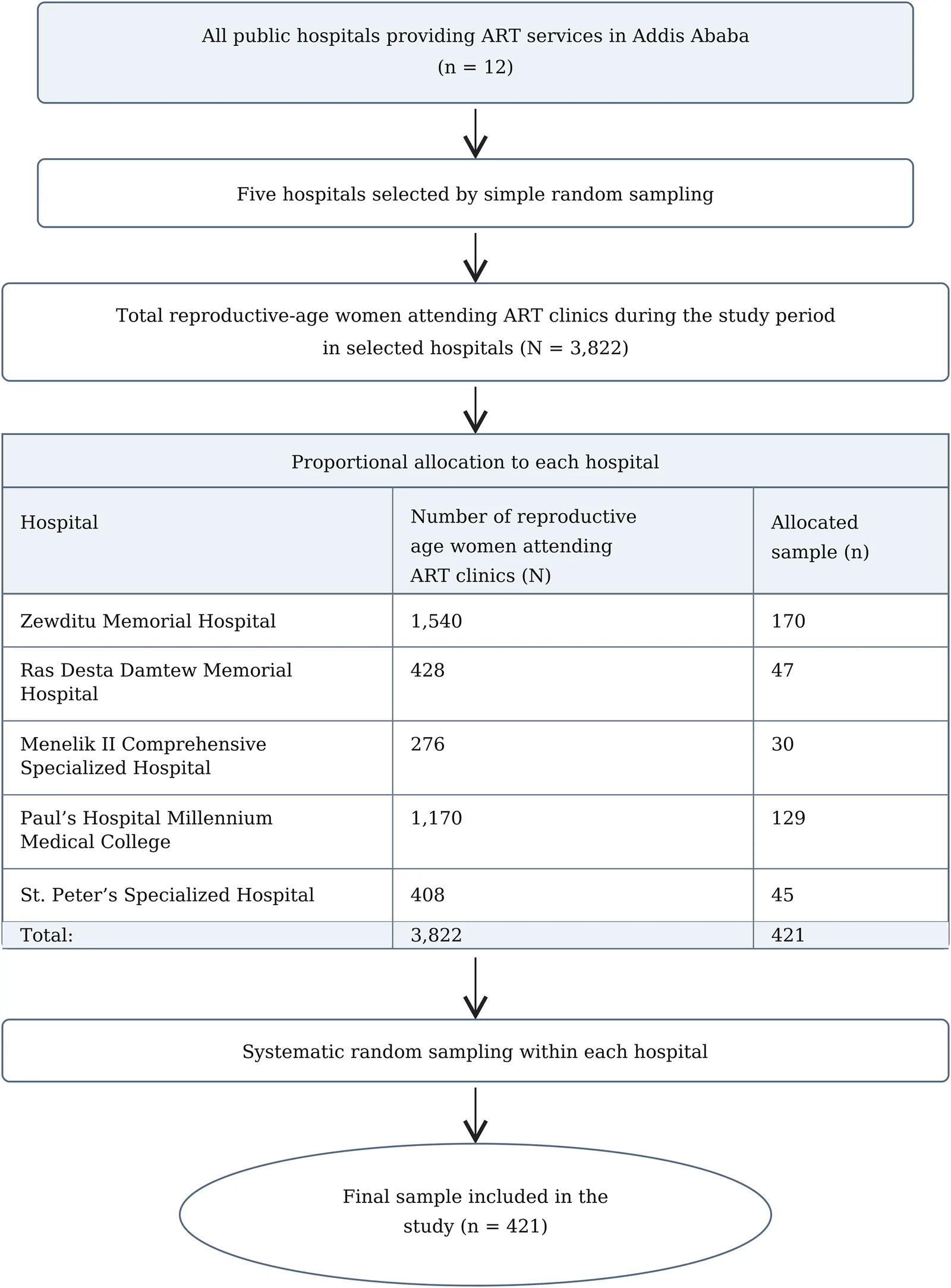
Sampling procedure for the study of unintended pregnancy and associated factors among women on ART in selected public hospitals, Addis Ababa, Ethiopia, 2025.

#### Qualitative component.

**Sample size determination and sampling.** Participants were purposively selected to achieve data saturation. Fourteen participants were included: eight HIV-positive women, three health professionals, and three case managers/adherence supporters from ART units. These participant categories were selected to capture diverse perspectives related to unintended pregnancy experiences, counseling, and ART service delivery. Women were recruited to explore their lived experiences, while health professionals and case managers were included to provide complementary perspectives on counseling and support services. Selection considered diversity in age, marital status, education, professional background, and years of ART service experience. HIV-positive women with a history of unintended pregnancy were identified during ART visits and recruited after providing informed consent. Recruitment continued until saturation was achieved across the participant groups, where additional participants did not provide new information relevant to the study objectives.

### Variables

#### Dependent variable.

Unintended pregnancy, defined as a pregnancy reported as mistimed or unwanted (1 = unintended, 0 = intended).

#### Independent variables.

Independent variables included socio-demographic factors (age, educational status, occupation, marital status, monthly family income, and partner’s educational status); HIV-related factors (duration since HIV diagnosis, CD4 count, disclosure of HIV status to partner, and knowledge of MTCT/PMTCT); family planning-related factors (contraceptive use after HIV diagnosis and exposure to sexual and reproductive health counseling); and sexual behavior factors (number of sexual partners in the past year).

### Operational definition

**Unintended pregnancy:** A pregnancy reported the participant as either mistimed (occurred earlier than desired) or unwanted (not desired at all) at the time of conception [3].

**HIV status disclosure:** Participant’s self-report of having disclosed her HIV-positive status to her sexual partner (Yes/No).

**Knowledge of MTCT and PMTCT:** Measured using 11 items on HIV transmission, timing of mother-to-child transmission, and prevention methods. Correct responses were scored as 1 and incorrect as 0 (range: 0–11). Based on the demarcation formula, participants scoring ≥6 were categorized as having “*good knowledge*” and ≤5 as “*poor knowledge*” [43].

**Contraceptive use after HIV diagnosis:** Self-reported use of any modern contraceptive method (such as injectable contraceptives, oral contraceptive pills, implants, intrauterine devices, or male condoms) at any time after being diagnosed with HIV (Yes/No).

**Sexual and reproductive health (SRH) counseling exposure:** Assessed using four yes/no items on receipt of counseling regarding contraceptive methods, unintended pregnancy, pregnancy and childbearing, or other SRH issues from ART providers. Each “Yes” was scored as 1 and “No” as 0. Total scores ranged from 0 to 4. Using the demarcation formula *((highest –* lowest)/2) + *lowest*, participants scoring ≥2 were classified as having “*adequate SRH counseling exposure*” and <2 as “*inadequate SRH counseling exposure.*”

### Data collection tool and procedures

Quantitative data were collected using a structured, interviewer-administered questionnaire adapted from previous studies [20,25,27–29,31,42,44,45], designed to capture all study variables. The questionnaire included sections on socio-demographic characteristics, contraceptive use, HIV-related factors and knowledge of MTCT/PMTCT, pregnancy and childbearing counseling, sexual and reproductive health behaviors, and reproductive characteristics such as future fertility desire. Clinical and medical history variables, including CD4 count, time since HIV diagnosis, and duration on ART, were extracted from patient records using a standardized checklist. Data collection was carried out by five trained BSc nurses under the supervision of a public health officer, with oversight from the principal investigator.

Qualitative data were collected through in-depth interviews using a semi-structured interview guide, developed specifically by the authors based on the study objectives and a review of relevant literature to explore experiences, perceptions, barriers, and strategies related to unintended pregnancy among women receiving ART. The guide is provided as S1 Text. It was prepared in English, translated into Amharic, and back-translated to ensure linguistic and cultural accuracy. Two trained MPH professionals conducted face-to-face interviews in private rooms after obtaining participants’ written informed consent. Interviews were audio-recorded, lasted 25–40 minutes on average, and continued until thematic saturation was reached.

### Data quality assurance

Data quality was ensured through pre-testing the quantitative questionnaire on 5% of the sample, with subsequent adjustments to question clarity and order. Data collectors received two days of training on study objectives, interview techniques, and confidentiality. Supervisors and the principal investigator monitored daily data collection for completeness and consistency. For qualitative data, the interview guide was pre-tested, and transcripts were verified by advisors and experienced qualitative researchers. Purposeful sampling with variation was used to capture diverse perspectives.

### Data processing and analysis

Quantitative data were first checked for completeness, then coded and entered into EpiData version 7.2.6 before being exported to Stata version 17 for further cleaning and analysis. Descriptive statistics such as medians, interquartile ranges, frequencies, and percentages were calculated and presented using tables and figures.

Prior to regression analysis, assumptions for logistic regression were checked. Multicollinearity was not a concern (mean variance inflation factor (VIF) = 1.23; all VIF < 10), and since all variables were categorical, the linearity assumption was inherently satisfied. Bivariable logistic regression was conducted to identify candidate variables (p < 0.25) for inclusion in the multivariable model. Variables meeting this criterion were adjusted for in the multivariable logistic regression model to control for potential confounding and identify independent factors associated with unintended pregnancy. Adjusted odds ratios (AORs) with 95% confidence intervals were computed to identify factors associated with unintended pregnancy. Model fit was assessed using the Hosmer–Lemeshow test (χ^2^ = 315.76, p = 0.217), and statistical significance was set at p < 0.05.

For the qualitative data, interviews were transcribed verbatim and translated into English. Thematic analysis was conducted using NVivo version 12. The transcripts were coded inductively by the first author using NVivo version 12 and subsequently organized into categories and overarching themes through an iterative process. To enhance the trustworthiness of the analysis, the coding framework, emerging categories, and themes were reviewed and discussed with the co-authors, and feedback from these discussions was incorporated to refine the coding framework and achieve consensus on the interpretation of the themes. Representative quotations were selected to illustrate the findings. The qualitative component of this study is reported in accordance with the Consolidated Criteria for Reporting Qualitative Research (COREQ) guideline.

Following separate analyses of the quantitative and qualitative data, the findings were integrated during interpretation using a triangulation approach. Areas of convergence, complementarity, and divergence between the two datasets were examined to generate meta-inferences and provide a comprehensive understanding of unintended pregnancy among women receiving ART.

## Results

### Quantitative findings

#### Socio-demographic characteristics of participants.

A total of 421 HIV-positive women were approached, of whom 384 participated in the quantitative survey (response rate: 91.21%). The mean age of participants was 34 years (SD ± 7.07), with the largest proportion aged 25–34 years (197, 51.30%). Regarding educational status, 133 (34.64%) had no formal education, while 139 (36.20%) had completed primary education. The majority of participants were married, 228 (59.38%), and 222 (57.81%) were employed. Detailed socio-demographic characteristics are presented in Table 1.

**Table 1:** Socio-demographic characteristics of HIV-positive reproductive-age women attending ART clinics in selected public hospitals, Addis Ababa, Ethiopia, 2025 (n = 384).

Variables	Categories	Number	Percent
Age	15–24 years	24	6.25%
	25–34 years	197	51.30%
	35–49 years	163	42.45%
Educational status of the women	No formal education	133	34.64%
	Primary education	139	36.20%
	Secondary education	91	23.70%
	Higher education	21	5.47%
Occupational status of the women	Not formally employed	162	42.19%
	Employed	222	57.81%
Marital status	Single	71	18.49%
	Married	228	59.38%
	Divorced	70	18.23%
	Widowed	15	3.91%
Family income	<5000 ETB	83	21.61%
	5000 - 10000 ETB	233	60.68%
	≥10000 ETB	68	17.71%
Partner’s educational status	No formal education	86	22.40%
	Primary education	137	35.68%
	Secondary education	128	33.33%
	Higher education	33	8.59%

#### HIV-related characteristics.

The majority of participants, 303 (78.91%), had known their HIV status for five or more years, with a median duration of 6 years (IQR: 5–9). Similarly, 339 (88.28%) had been on ART for at least three years, with a median treatment duration of 5 years (IQR: 4–7). Most women 347 (90.36%) had a recent CD4 count ≥200 cells/µL, with a median CD4 count of 359 cells/µL (IQR: 286–458). Disclosure of HIV status to partners was reported by 276 (71.88%) participants, and 246 (64.06%) indicated that their partner had been tested for HIV. Among partners who were tested, 205 (83.33%) were HIV-positive, and 173 (84.39%) of these partners were currently receiving ART (Table 2).

**Table 2:** HIV diagnosis and treatment-related characteristics of HIV-positive reproductive-age women attending ART clinics in selected public hospitals in Addis Ababa, 2025 (n = 384).

Variable	Categories	Number	Percent
Time since HIV diagnosis (n = 384)	< 5 years	81	21.09%
	≥ 5 years	303	78.91%
ART treatment duration (n = 384)	< 3 years	45	11.72%
	≥ 3 years	339	88.28%
CD4 count category (n = 384)	< 200	37	9.64%
	≥200	347	90.36%
HIV status disclosure to partner (n = 384)	Yes	276	71.88%
	No	108	28.13%
Partner tested for HIV (n = 384)	Yes	246	64.06%
	No	138	35.94%
Partner HIV test result (n = 246)	HIV-positive	205	83.33%
	HIV-negative	41	16.67%
Partner on ART (n = 205)	Yes	173	84.39%
	No	18	8.78%
	I do not know	14	6.83%

Overall, 306 (79.69%) participants were classified as having good knowledge of MTCT/PMTCT. Most women, 294 (76.56%), were aware that HIV can be transmitted from mother to child. Regarding the timing of transmission, 267 (69.53%) recognized transmission during pregnancy, 287 (74.74%) during delivery, and 279 (72.66%) during breastfeeding. Detailed information on knowledge of MTCT and its prevention methods is presented in Table 3.

**Table 3:** Knowledge of MTCT and PMTCT among HIV-positive women of reproductive age attending ART clinics in selected public hospitals, Addis Ababa, Ethiopia, 2025 (n = 384).

Variables	Categories		Number	Percent
Knowledge of HIV transmission modes	Sexual intercourse	Yes	363	94.53%
		No	21	5.47%
	Mother-to-child transmission	Yes	294	76.56%
		No	90	23.44%
	Sharp metal injury	Yes	308	80.21%
		No	76	19.79%
	Blood transfusion	Yes	163	42.45%
		No	221	57.55%
Knowledge of timing of MTCT	During pregnancy	Yes	267	69.53%
		No	117	30.47%
	During delivery	Yes	287	74.74%
		No	97	25.26%
	During breastfeeding	Yes	279	72.66%
		No	105	27.34%
Knowledge of methods to prevent MTCT	Prevent unintended pregnancy	Yes	148	38.54%
		No	236	61.46%
	Use of ARVs	Yes	266	69.27%
		No	118	30.73%
	Cesarean section delivery	Yes	142	36.98%
		No	242	63.02%
	Exclusive breastfeeding	Yes	248	64.58%
		No	136	35.42%
Overall knowledge of MTCT/PMTCT	Good knowledge		306	79.69%
	Poor knowledge		78	20.31%

#### Family planning practices.

Most participants, 242 (63.0%), reported using a family planning method after HIV diagnosis. Among contraceptive users (n = 242), only 47 (19.4%) obtained contraceptives from ART units, while the majority accessed them from hospital family planning units (96, 39.7%) or other health facilities (53, 21.9%). The remaining participants obtained contraceptives from pharmacies (38, 15.7%) and non-governmental organization clinics, including the Family Guidance Association (8, 3.3%). When asked about the integration of family planning services into ART units, most women supported this approach, with 185 (48.2%) supporting and 98 (25.5%) strongly supporting integration, while 25 (6.5%) opposed, 6 (1.6%) strongly opposed, and 70 (18.2%) were neutral. Among the 142 women (37.0%) who were not using contraception, the main reasons reported were concerns about side effects (40, 28.2%), partner disapproval (25, 17.6%), and confidentiality concerns (22, 15.5%) (Fig 2).

**Fig 2:**
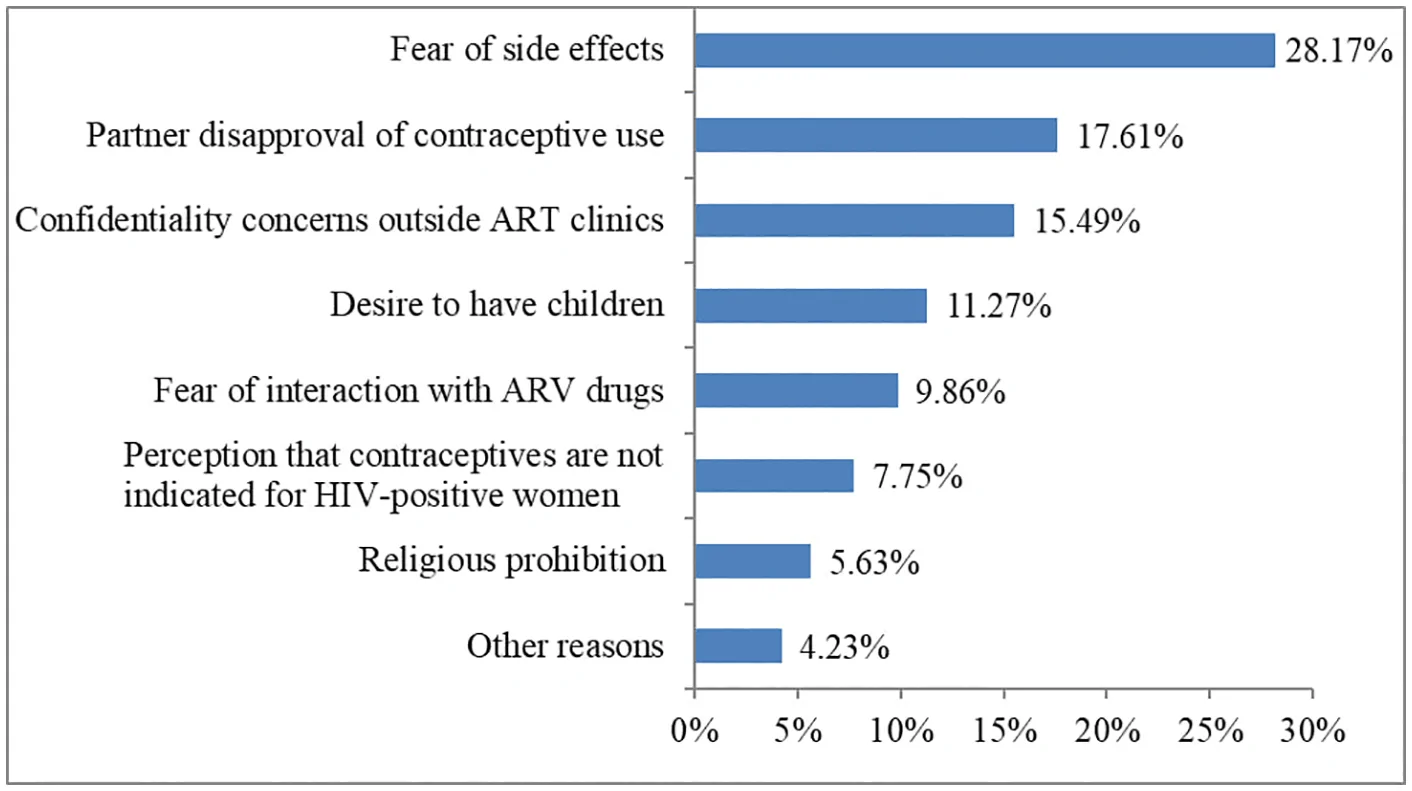
Reasons for non-use of contraceptive methods among HIV-positive reproductive-age women attending ART clinics in selected public hospitals, Addis Ababa, Ethiopia, 2025 (n = 142).

#### Sexual and reproductive health counseling characteristics.

Although 347 (90.4%) participants reported a need to discuss sexual and reproductive health (SRH) issues with providers, only 157 (40.9%) had received adequate counseling. Counseling coverage varied by topic: 139 (36.2%) received contraceptive counseling, 74 (19.3%) received counseling on unintended pregnancy, 156 (40.6%) received pregnancy and childbearing counseling, and 164 (42.7%) received counseling on other sexual health issues (Table 4).

**Table 4:** Sexual and reproductive health counseling received by HIV-positive reproductive-age women attending ART clinics in selected public hospitals, Addis Ababa, Ethiopia, 2025 (n = 384).

Variables	Categories	Number	Percent
Need to discuss SRH issues with providers	Yes	347	90.4%
	No	37	9.6%
Counseling on contraceptives	Yes	139	36.2%
	No	245	63.8%
Counseling on unintended pregnancy	Yes	74	19.3%
	No	310	80.7%
Counseling on pregnancy and childbearing	Yes	156	40.6%
	No	228	59.4%
Counseling on sexual health issues	Yes	164	42.7%
	No	220	57.3%
Overall SRH counseling exposure	Adequate	157	40.9%
	Inadequate	227	59.1%

#### Sexual behavior characteristics.

A total of 131 (34.1%) participants reported initiating sexual intercourse before the age of 18 years. All participants reported being sexually active in the past year, of whom 151 (39.3%) had two or more sexual partners during this period. Condom use at last sexual intercourse was low; only 107 (27.9%) participants reported using a condom, while 250 (65.1%) did not use a condom and 27 (7.0%) could not recall whether a condom was used. Among those who did not use condoms, the most commonly reported reasons were partner disapproval (30.8%) and personal dislike of condoms (30.0%), followed by lack of knowledge about where to obtain condoms (12.4%), having an HIV-positive partner (12.0%), and the desire to have children (10.4%) (Table 5).

**Table 5:** Sexual characteristics of HIV-positive reproductive-age women attending ART clinics in selected public hospitals, Addis Ababa, Ethiopia, 2025 (n = 384).

Variables	Categories	Number	Percent
Age at first sexual intercourse	< 18 years	131	34.11%
	≥ 18 years	253	65.89%
Number of sexual partners in the past year	One partner	233	60.68%
	Two or more partners	151	39.32%
Condom use at last sexual intercourse	Yes, used a condom	107	27.86%
	No, I did not use a condom	250	65.10%
	Do not remember	27	7.03%
Reasons for not using a condom	Partner disapproved	77	30.80%
	Did not like using condoms	75	30.00%
	Did not know where to get condoms	31	12.40%
	The partner is also HIV-positive	30	12.00%
	Desiring pregnancy	26	10.40%
	Other reasons	11	4.40%

#### Reproductive characteristics and prevalence of unintended pregnancy.

All participants had experienced at least one pregnancy after their HIV diagnosis. Of the most recent pregnancies, 149 (38.8%; 95% CI: 34.0–43.8%) were unintended, including 45 (30.2%) unwanted and 104 (69.8%) mistimed pregnancies, while 235 (61.2%) were intended. Regarding pregnancy outcomes, 238 (62.0%) resulted in live births, 70 (18.2%) in medical abortions, and 52 (13.5%) in spontaneous abortions. Among last-born children, 348 (90.63%) were HIV-negative, while 26 (6.8%) were HIV-positive. More than half of the participants (58.9%) expressed a desire for future childbearing, with most preferring one to two children (Table 6).

**Table 6:** Reproductive characteristics of HIV-positive reproductive-age women attending ART clinics in selected public hospitals, Addis Ababa, Ethiopia, 2025 (n = 384).

Variables	Categories	Number	Percent (%)
Number of pregnancies after knowing HIV status	1–2 pregnancies	308	80.2%
	≥ 3 pregnancies	76	19.8%
Outcome of the most recent pregnancy	Live birth	238	62.0%
	Medical abortion	70	18.2%
	Spontaneous abortion	52	13.5%
	Stillbirth	24	6.3%
HIV status of live-born child (n = 238)	HIV-negative	220	92.4%
	HIV-positive	11	4.6%
	Not confirmed	7	2.9%
Desire for future childbearing	Yes	226	58.9%
	No	158	41.1%
Desired number of future children (n = 226)	1-2 children	200	88.5%
	≥ 3 children	26	11.5%

#### Factors associated with unintended pregnancy.

In the bivariable logistic regression analysis, variables with a p-value <0.25 were considered for inclusion in the multivariable logistic regression model. Accordingly, age, women’s educational status, occupational status, partner’s educational status, duration since HIV diagnosis, HIV status disclosure to partners, knowledge of MTCT/PMTCT, contraceptive use after HIV diagnosis, SRH counseling exposure, and number of sexual partners in the past year were entered into the multivariable model. In the multivariable logistic regression analysis, women’s educational status, duration since HIV diagnosis, HIV status disclosure to partners, contraceptive use after HIV diagnosis, and SRH counseling exposure were significantly associated with unintended pregnancy. The adjusted odds ratios, 95% confidence intervals, and p-values for both significant and non-significant variables included in the final model are presented in Table 7.

**Table 7:** Factors associated with unintended pregnancy among women attending ART clinics in selected public hospitals, Addis Ababa, 2025 (n = 384).

Variables	Categories	Unintended pregnancy		COR (95% CI)	AOR (95% CI)	P value
		Yes (%)	No (%)			
Age	15-24 years	4(16.7)	20(83.3)	0.32(0.10-0.97)	0.42(0.11-1.65)	0.21
	25-34 years	82(41.6)	115(58.4)	1.13(0.74-1.73)	1.53(0.90-2.60)	0.11
	35-49 years	63(38.7)	100(61.3)	1	1	
Women’s educational status	No formal education	82(61.7)	51(38.3)	1	1	
	Primary education	44(31.7)	95(68.3)	0.29(0.17-0.47)	0.31(0.17-0.56)*	<0.01
	Secondary education	21(23.1)	70(76.9)	0.19(0.10-0.34)	0.24(0.11-0.51)*	<0.01
	Higher education	2(9.5)	19(90.5)	0.07(0.01-0.29)	0.11(0.02-0.66)*	0.02
Women’s occupational status	Not formally employed	56(34.6)	106(65.4)	0.73(0.48-1.11)	0.9(0.53-1.52)	0.69
	Employed	93(41.9)	129(58.1)	1	1	
Partner’s educational status	No formal education	37(43.0)	49(57.0)	1	1	
	Primary education	60(43.8)	77(56.2)	1.03(0.60-1.78)	1.38(0.72-2.65)	0.34
	Secondary education	46(35.9)	82(64.1)	0.74(0.42-1.30)	1.73(0.85-3.52)	0.13
	Higher education	6(18.2)	27(81.8)	0.29(0.11-0.79)	1.16(0.33-4.07)	0.82
Duration since HIV diagnosis	< 5 years	21(25.9)	60(74.1)	1	1	<0.01
	≥ 5 years	128(42.2)	175(57.8)	2.09(1.21-3.61)	2.77(1.44-5.34)*	
Disclosure status to partners	Yes	96(34.8)	180(65.2)	1	1	0.02
	No	53(49.1)	55(50.9)	1.81(1.15-2.84)	1.92(1.11-3.31)*	
Knowledge of MTCT/PMTCT	Good knowledge	113(36.9)	193(63.1)	1	1	0.95
	Poor knowledge	36(46.2)	42(53.8)	1.46(0.89-2.42)	1.02(0.56-1.87)	
Contraceptive use after HIV diagnosis	Yes	60(24.8)	182(75.2)	1	1	<0.01
	No	89(62.7)	53(37.3)	5.09(3.25-7.97)	4.77(2.85-8.04)*	
SRH counseling exposure	Adequate counseling	37(23.6)	120(76.4)	1	1	0.03
	Inadequate counseling	112(49.3)	115(50.7)	3.16(2.01-4.96)	1.86(1.08-3.20)*	
Number of sexual partners in the past year	1 partner	83(35.6)	150(64.4)	1	1	0.40
	≥ 2 partners	66(43.7)	85(56.3)	1.40(0.92-2.13)	1.26(0.74-2.12)	

Note: Variables with p-value <0.25 in the bivariable analysis were entered into the multivariable logistic regression model. Variables with p-value ≥0.05 in the final model were retained but were not statistically significantly associated with unintended pregnancy. * Significant at p < 0.05; AOR = Adjusted Odds Ratio; COR = Crude Odds Ratio; CI = Confidence Interval.

Compared with women who had no formal education, those with primary, secondary, and higher education were 69%, 76%, and 89% less likely to experience unintended pregnancy, respectively (AOR = 0.31; 95% CI: 0.17–0.56; AOR = 0.24; 95% CI: 0.11–0.51; and AOR = 0.11; 95% CI: 0.02–0.66). Women diagnosed with HIV for five or more years were 2.77 times more likely to experience unintended pregnancy compared with those diagnosed within the past five years (AOR = 2.77; 95% CI: 1.44–5.34). Similarly, women who had not disclosed their HIV status to their partners were 92% more likely to experience unintended pregnancy compared with those who had disclosed their status (AOR = 1.92; 95% CI: 1.11–3.31). Women who did not use contraceptives after HIV diagnosis were 4.77 times more likely to experience unintended pregnancy compared with those who used contraceptives (AOR = 4.77; 95% CI: 2.85–8.04). In addition, women with inadequate exposure to sexual and reproductive health counseling were 86% more likely to experience unintended pregnancy compared with those who received adequate counseling (AOR = 1.86; 95% CI: 1.08–3.20).

### Qualitative findings

Fourteen participants were interviewed for the qualitative component: eight HIV-positive women who had experienced unintended pregnancy, three healthcare providers (two nurses, one medical doctor), and three case managers/adherence supporters. HIV-positive women participants ranged in age from 25 to 37 years and represented diverse marital statuses (single, married, and widowed) and educational backgrounds.

Four main themes emerged from the qualitative analysis: (1) reasons for unintended pregnancy, (2) barriers to contraceptive use, (3) inadequacy of SRH counseling, and (4) strategies for prevention.

#### Theme 1: Reasons for unintended pregnancy.

Participants described multiple intersecting factors contributing to unintended pregnancy. A prominent sub-theme was negligence and diminished self-worth among women living with HIV. As one nurse explained.

*"HIV-positive women exhibit distinct behavioral patterns compared to the general population, potentially stemming from the prolonged use of ART or the profound psychological impact of their diagnosis. These women often experience a sense of marginalization and neglect... focusing exclusively on ARV medications while overlooking their comprehensive SRH needs." (34-year-old nurse)*

Another recurring reason was inadequate counseling and knowledge gaps. A 31-year-old single woman described the following:

*"I experienced an unintended pregnancy following a single encounter with an individual of unknown HIV status... The lack of adequate sexual and reproductive health counseling was a decisive factor; had I been empowered with the necessary preventative mechanisms and counseling prior to this event, this pregnancy would almost certainly not have occurred."*

#### Theme 2: Barriers to contraceptive use.

Consistent with quantitative findings, participants identified fear of side effects and drug interactions as major barriers. A 37-year-old married woman stated:

*“I am not using a contraceptive method due to fear of ARV drug interaction with the family planning methods. I operated under the misconception that family planning methods would antagonize my antiretroviral therapy, thereby compromising its therapeutic efficacy.”*

Partner disapproval emerged as another critical barrier, often intertwined with economic dependency:

*“My non-use of contraception is primarily driven by spousal opposition; specifically, my partner refuses to use condoms consistently. Because of this, I fear relationship discontinuation... and I rely on him for financial support." (25-year-old widowed woman)*

A significant systems-level barrier was confidentiality concerns related to service fragmentation. A case manager with 13 years of experience noted:

*“The primary deterrent identified was confidentiality apprehensions, specifically a profound reluctance to access separate family planning services due to fear of status disclosure. It is evident that a critical deficit in service integration exists within ART units.*

#### Theme 3: Inadequate sexual and reproductive health counseling.

Both providers and clients acknowledged that SRH counseling is suboptimal and inconsistent. An adherence supporter explained:

*“Despite the high number of HIV-positive individuals and our limited staff, we strive to provide as much support as possible. Currently, we prioritize adolescents, young adults, and women facing significant adherence challenges." (36-year-old adherence supporter)*

A 29-year-old married woman described her experience:

*“I have noticed that counseling is not provided regularly. My initial session was excellent... However, since that first time, no one has offered me further counseling. I believe ongoing support is necessary.”*

Participants also reported disrespectful provider interactions that discouraged help-seeking:

*“On one occasion, a nurse shouted at me simply for asking for condoms. It seems that staff at the ART clinic are often overly focused on dispensing ARV medications, neglecting the other essential health services and respectful care they are supposed to provide." (25-year-old single woman)*

#### Theme 4: Strategies for prevention.

Participants proposed several interventions. The most frequently mentioned was the integration of family planning into ART services:

*“Strengthening the integration of family planning services within ART units is essential for increasing contraceptive uptake among women living with HIV." (40-year-old case manager)*

A medical doctor recommended systems-level improvement:

*“Incorporating and strengthening sexual and reproductive health education and counseling as one package service... Assigning additional ART providers... is also good, in order to give adequate and appropriate SRH counseling, since there is a high client flow." (32-year-old medical doctor)*

Women themselves requested comprehensive health education:

*“Even I do not know the services provided in the ART treatment unit other than HAART service. Thus, SRH education in the waiting area daily through different ways should be given to us." (37-year-old HIV-positive woman)*

### Integration of quantitative and qualitative findings

The integration of quantitative and qualitative data provides a comprehensive understanding of unintended pregnancy among HIV-positive women. Key quantitative associations are explained and contextualized by qualitative findings (Table 8).

**Table 8:** Joint display of integrated quantitative and qualitative findings on unintended pregnancy and associated factors among women on ART in selected public hospitals, Addis Ababa, 2025.

Quantitative finding	Qualitative finding	Meta-Inference
Higher education reduced the odds of unintended pregnancy (AOR = 0.31, 0.24, and 0.11 for primary, secondary, and higher education).	Women with limited education reported misconceptions about contraceptive methods and difficulty negotiating reproductive decisions with partners.	Education improves understanding of contraceptive information and decision-making autonomy.
A longer duration since HIV diagnosis increased the odds of unintended pregnancy (AOR = 2.77; 95% CI: 1.44–5.34).	Providers noted that women stable on ART may focus mainly on medication adherence and overlook SRH needs.	Long-term ART care may reduce attention to reproductive health without continuous counseling.
Non-disclosure of HIV status to a partner increased the odds of unintended pregnancy (AOR = 1.92; 95% CI: 1.11–3.31).	Women reported fear of relationship conflict and financial dependency, limiting communication about contraception.	Non-disclosure creates power imbalances that hinder joint reproductive decision-making.
Non-use of contraception increased the odds of unintended pregnancy (AOR = 4.77; 95% CI: 2.85–8.04).	Women reported fear of side effects, misconceptions about ARV–contraceptive interaction, partner opposition, and confidentiality concerns when accessing FP services.	Contraceptive non-use is influenced by psychosocial fears, partner dynamics, and fragmented service delivery.
Inadequate SRH counseling increased the odds of unintended pregnancy (AOR = 1.86; 95% CI: 1.08–3.20).	Providers and women described SRH counseling as inconsistent due to heavy workloads and prioritization of ART services.	Weak SRH counseling within ART services contributes to unintended pregnancy risk.

The quantitative findings of non-use of contraception, low education, inadequate SRH counseling, longer duration of HIV, and non-disclosure to partners are supported and explained by the qualitative narratives. Women’s fears, partner dynamics, provider attitudes, and fragmented services provide the mechanisms behind unintended pregnancies. These findings emphasize the need for integrated, client-centered interventions that address both behavioral and system-level barriers.

## Discussion

This mixed-methods study assessed the prevalence and factors associated with unintended pregnancy among HIV-positive women attending ART units in public hospitals in Addis Ababa, Ethiopia. The integration of quantitative and qualitative findings provides a comprehensive understanding of the multidimensional factors associated with unintended pregnancy in this population.

The prevalence of unintended pregnancy among HIV-positive women in this study was 38.8%, with 30.2% unwanted and 69.8% mistimed. This finding is consistent a recent systematic review and meta-analysis from East Africa reporting a pooled prevalence of 40.98% among women living with HIV, with a pooled prevalence of 28.38% in Ethiopia [15], as well as studies conducted in Enugu, Nigeria (37.2%) [24], and Oromia Regional State, Ethiopia (39.8%) [46]. However, the prevalence was higher than that reported in Bahir Dar, Ethiopia (22.4%) [31], possibly due to differences in study populations, as the Bahir Dar study included only HIV-positive pregnant women enrolled in PMTCT programs, potentially excluding women who experienced unintended pregnancies and opted for termination. Conversely, the prevalence in the present study was lower than findings from South Africa (52%) [28] and Botswana (44%) [29], which may reflect differences in healthcare systems, contraceptive method mix, socioeconomic contexts, and study periods.

Notably, the 38.8% prevalence persists despite increased availability of HIV services and awareness of contraception and MTCT/PMTCT. Qualitative findings explain this paradox: women reported inconsistent counseling, fear of side effects, partner disapproval, and confidentiality concerns as barriers to effective contraceptive use. As one participant stated, “*the counseling given to me is too irregular and not adequate and often narrowly restricted to contraceptive utilization*” (27-year-old woman). This highlights that service availability alone is insufficient without quality counseling and addressing psychosocial barriers.

Among women who experienced unintended pregnancy, 18.2% reported undergoing a medical abortion. Although this study did not assess access to abortion services, this finding underscores the importance of comprehensive reproductive health care for women living with HIV, including timely referral to safe abortion services where legally available. It also highlights the need to strengthen contraceptive counseling and access to effective family planning methods to reduce unintended pregnancies. In addition, integrating post-abortion family planning counseling and contraceptive services into routine HIV care may help prevent subsequent unintended pregnancies.

Educational status was found to be protective against unintended pregnancy. Women with primary, secondary, and higher education had 69%, 76%, and 89% lower odds, respectively, of experiencing unintended pregnancy compared with women with no formal education. This finding is consistent with studies conducted in Switzerland [18] and East Africa [15]. The qualitative findings further indicated that limited education may be associated with misconceptions about contraceptive methods and difficulties in negotiating reproductive decisions. One participant explained, “*my contraceptive hesitancy is compounded by perceived risks of the side effects of family planning since I have no formal education.*” Education likely empowers women to understand contraceptive information, navigate health services, and make informed reproductive choices.

Women who had lived with HIV for five or more years had 2.77 times higher odds of unintended pregnancy compared with those more recently diagnosed. This finding is consistent with studies conducted in the United States [19] and northern Uganda [47]. As women stabilize on ART and health improves, fertility desires may increase [48]. Healthcare providers in this study noted that women on long-term ART sometimes become “*disengaged... focusing exclusively on ARV medications while overlooking comprehensive SRH needs.”* Without adequate preconception counseling and contraceptive support, increased fertility desire may translate into unintended pregnancies. These findings highlight the importance of continuously integrating sexual and reproductive health services into long-term HIV care.

Non-disclosure of HIV status to partners was associated with nearly two-fold higher odds of unintended pregnancy, consistent with findings from referral hospitals in the Amhara Regional State [49]. Disclosure facilitates partner support for contraceptive use and promotes joint reproductive decision-making. As one participant described, non-disclosure created communication barriers that prevented couples from discussing family planning together. Healthcare providers should therefore encourage and support safe disclosure practices, recognizing that disclosure is a gradual process that requires appropriate counseling and support [50].

Non-use of contraception after HIV diagnosis increased the odds of unintended pregnancy by 4.77 times, consistent with findings from Zimbabwe [30]. Qualitative findings provided insights into the reasons for contraceptive non-use. Women expressed fears of side effects, concerns about potential interactions between contraceptive methods and antiretroviral drugs, partner opposition, and reluctance to seek family planning services due to stigma. A 37-year-old married woman stated, “*I am not using a contraceptive method due to fear of ARV drug interaction... I operated under the misconception that family planning methods would antagonize my antiretroviral therapy.*” Partner dynamics also played a significant role in contraceptive decision-making. As another participant explained, “*my partner refuses to use condoms consistently... I fear relationship discontinuation... and I rely on him for financial support*” (25-year-old widowed woman). These findings underscore the need for counseling that addresses misconceptions, partner engagement, and relationship power dynamics, in addition to contraceptive provision.

Only 19.4% of women obtained contraceptives from ART units, although 73.7% supported integrating family planning services within ART clinics. Qualitative findings help explain this discrepancy. A case manager noted, *“The primary deterrent was confidentiality concerns, particularly fear of status disclosure when accessing separate family planning services. There is clearly a deficit in service integration within ART units.*” Similarly, a 32-year-old woman described stigma-related barriers that discouraged her from seeking services in separate units. Integrating family planning services into ART clinics could reduce confidentiality concerns, minimize service fragmentation, decrease the burden of multiple clinic visits, and promote comprehensive care. These findings align with previous studies documenting the negative impact of fragmented services on reproductive health outcomes among women living with HIV [51].

Condom use at the last sexual intercourse was low (27.9%), despite its critical role in providing dual protection against unintended pregnancy and sexually transmitted infections. The reasons for non-use identified in this study, including partner disapproval, personal dislike of condoms, misconceptions among women with HIV-positive partners, and the desire to conceive, indicate that condom use is influenced by behavioral, interpersonal, and informational factors. These findings were further supported by the qualitative interviews, which also highlighted inadequate sexual and reproductive health counseling and relationship dynamics as barriers to consistent condom use. Strengthening dual protection counseling within ART services, addressing misconceptions, involving male partners where appropriate, and integrating comprehensive sexual and reproductive health counseling into routine HIV care may improve condom uptake and may improve consistent condom use and strengthen HIV prevention and prevention of mother-to-child transmission (PMTCT) efforts.

Finally, inadequate sexual and reproductive health counseling was associated with an 86% higher likelihood of unintended pregnancy, consistent with findings from Illu-Ababora, Southwest Ethiopia [42]. Qualitative findings revealed systemic gaps in counseling provision due to high client volumes and limited healthcare staff. Providers acknowledged that counseling is often “*irregular*” and “*suboptimal*.” Women also reported feeling uncomfortable discussing their sexual and reproductive health needs and, in some cases, experiencing disrespectful care. One participant described it as “*On one occasion, a nurse shouted at me simply for asking for condoms*” (25-year-old woman). These findings highlight that the quality of counseling—not merely its availability—is crucial. Counseling services should comprehensively address contraceptive options, preconception care, and MTCT/PMTCT information and provide a safe, respectful, and non-judgmental environment for open discussion.

### Strengths and limitations of the study

This study has several methodological strengths. The use of a mixed-methods design enabled triangulation of quantitative results with qualitative insights, enhancing the depth and comprehensiveness of the findings. The inclusion of multiple public hospitals and the application of systematic sampling improved the representativeness and internal validity of the study. However, the cross-sectional design limits the ability to infer causal relationships between unintended pregnancy and associated factors. Furthermore, the reliance on self-reported data may have introduced recall and social desirability bias, particularly given the sensitive nature of reproductive health questions.

### Conclusions and recommendations

The prevalence of unintended pregnancy among women receiving ART in public hospitals in Addis Ababa was high. Unintended pregnancy was more common among women with no formal education, longer duration since HIV diagnosis, non-disclosure of HIV status to partners, non-use of contraception, and inadequate sexual and reproductive health counseling. Considering these associated factors may help strengthen the integration of family planning and reproductive health services within ART clinics and support the reproductive intentions of women living with HIV.

To address factors associated with unintended pregnancy, healthcare providers should offer routine, comprehensive, and non-judgmental counseling that addresses fertility intentions, contraceptive options, and HIV status disclosure. Male partner engagement strategies, including couples counseling, supported partner notification, and interventions that promote partner communication and shared decision-making, should be considered to address challenges related to HIV status disclosure and partner-related barriers identified in this study. Health authorities should strengthen the monitoring and evaluation of integrated HIV and reproductive health services in line with national HIV/AIDS priorities. Governmental and non-governmental organizations should support capacity building for healthcare providers and ensure the consistent availability of family planning methods. Future research using longitudinal and community-based designs is recommended to better understand causal relationships and population-level dynamics.

## Supporting information

S1 Text English version of the interview guide used for the qualitative interviews. (PDF)

## Abbreviations

ART: Antiretroviral therapy
ARV: Antiretroviral
AOR: Adjusted odds ratio
CI: Confidence interval
CD4: Cluster of Differentiation 4
COR: Crude odds ratio
ETB: Ethiopian Birr
HIV: Human Immunodeficiency Virus
MTCT: Mother-to-Child transmission
PMTCT: Prevention of mother-to-child transmission
SD: Standard deviation
SRH: Sexual and reproductive health
VIF: Variance inflation factor
