# Supplementary material for: Unintended pregnancy and associated factors among women on antiretroviral therapy in selected public hospitals, Addis Ababa, Ethiopia: A mixed-method study
Source: PLOS Glob Public Health. 2026 Nov 12;6(11):e0007626. doi: 10.1371/journal.pgph.0007626 (PMC13759079; doi:10.1371/journal.pgph.0007626)
Supplement: Supplementary file 1 [file pgph.0007626.s001.pdf]

S1 Text: Qualitative interview guide (English version)

Section A: Introduction and consent

Hello, my name is _____________. I am part of a research team conducting a study on unintended pregnancy and sexual and reproductive health among women receiving antiretroviral therapy (ART).

The purpose of this interview is to understand your experiences, opinions, and challenges regarding pregnancy, family planning, and sexual and reproductive health services.

Your participation is voluntary. You may choose not to answer any question or withdraw from the interview at any time. All information you provide will be kept strictly confidential and used only for research purposes. No personal identifiers will be included in any reports or publications.

The interview will take approximately 30–45 minutes.

Do you agree to participate in this interview?

☐ Yes ☐ No

Signature of interviewer: _____________

Date: _____________

Section B: Background information

Date_______________________

- Age: ________
- Type of participant: ☐ Woman on ART ☐ Healthcare provider ☐ Case manager / adherence supporter
- Health facility: ____________________
- Marital status: _____________________
- Educational level: __________________
- Occupation: _______________________
- Start time: _____________________
- End time: ______________________

Section C: Interview questions

1. Community perceptions
   - How is HIV discussed within your community?
     → *Probe: perceptions, stigma, disclosure practices*
   - How is pregnancy among women living with HIV viewed in your community?
     → *Probe: acceptance, cultural beliefs, discrimination*
2. Reasons for unintended pregnancy
   - In your experience, what factors contribute to unintended pregnancy among women receiving ART?

     → *Probe: knowledge or information gaps, counseling or service-related issues, partner-related factors, social, economic, or psychological influences*
3. Contraceptive use
   - What are your views on family planning use among women living with HIV?
   - (For women) Are you currently using any contraceptive method? Could you explain why or why not?

- (For healthcare providers) What is your observation regarding contraceptive use among women receiving ART?

  → *Probe: access to family planning services, concerns about side effects, perceived interaction with ART, partner opposition, cultural or religious considerations*

4. Counseling experience
   - How would you describe the counseling provided by ART healthcare providers regarding sexual and reproductive health?

     → *Probe: frequency and adequacy of counseling, clarity and completeness of information, comfort and openness when discussing sensitive issues, provider attitude and respect*
5. Barriers to services
   - What challenges do women face in accessing or discussing sexual and reproductive health services in ART clinics?

     → *Probe: confidentiality concerns, stigma or discrimination, limited service availability, interactions with healthcare providers*
6. Suggestions for prevention
   - What strategies would you recommend to prevent unintended pregnancy among women receiving ART?

     → *Probe: improving counseling and education, increasing awareness of family planning, integrating reproductive health services into ART care, involving partners and community support*

Section D: Closing

Thank you very much for your time and contribution. Your responses will help inform interventions to improve reproductive health services for women living with HIV.
